# Substance use during pregnancy: impact on Colorado community hospital

**DOI:** 10.1186/s42238-020-00047-9

**Published:** 2020-11-11

**Authors:** Jacinda Heintzelman, Lisa Persons, Igor Melnykov

**Affiliations:** 1 Colorado State University-Pueblo, Pueblo, USA; 2 University of Minnesota-Duluth, Duluth, USA

**Keywords:** Pregnancy, Newborn, Substance use, Cannabis, Healthcare costs

## Abstract

**Background:**

Neonatal Abstinence Syndrome (NAS) leads to increased length of stay (LOS), which leads to increased healthcare costs, and can cause financial burdens for hospitals. The purpose of the study was to determine the impact of substance use by pregnant women on a Colorado (CO) community hospital after state legalization of recreational cannabis.

**Methods:**

Data were gathered retrospectively through the electronic health record at an inpatient facility and described 607 mothers and 419 newborns (total *N* = 1026) who tested positive for drugs (urinalysis or blood for mother and urine, meconium, or cord blood for newborns). Screening for drugs was at discretion of healthcare provider if mother reported use or newborn showed symptoms of NAS. The patients who were not screened or tested negative were excluded from consideration. Newborns exposed to cannabis were compared to those exposed to other drugs (opioids, methadone, cocaine, barbiturates, benzodiazepines, amphetamines) on costs of newborn hospitalization, based on type of newborn bed and length of stay (LOS). Group comparisons were done using Cochran-Armitage chi-square tests and two-sample t-tests.

**Results:**

The proportion of screened patients testing positive for illicit and prescribed substances increased significantly from 2013 (33.4%) to 2017 (50.2%) (*p* < 0.001). The LOS of drug-exposed newborns increased significantly over the years (*p* < 0.0001). Newborns testing positive for cannabis were more likely to remain in a normal newborn nursery (NSY) bed (69.8%) than those testing positive for other drugs (27.7%), with an average hospital LOS (4.6 days) significantly shorter (*p* < 0.001) than that of other-drug exposed newborns (14.2 days). Combined healthcare costs for other-drug exposed newborns ($23,495,221) were significantly higher (*p* < 0.001) than for cannabis-exposed newborns ($2,885,139); both groups had significantly higher costs (*p* < 0.001) than normal healthy newborns ($2,166,649).

**Conclusion:**

Drug-exposed newborns have a significant financial impact on hospital healthcare costs, largely due to more expensive bed placement and longer LOS.

## Background

The current use of illicit and prescribed substances in the United States (U.S.) is considered a national crisis, and pregnant women cannot be ignored in this situation since a newborn may be negatively affected through intrauterine exposure. Newborn exposure can lead to an increased length of stay (LOS) and an increase in healthcare costs when compared to a normal healthy newborn, and negatively affect hospitals. When a mother ingests addictive substances, the substance(s) moves across the placental barrier and can lead to Neonatal Abstinence Syndrome (NAS) or neonatal dependency (Young et al. 2015). Symptoms of NAS include excessive crying, tremors, sweating, poor sleep, increased muscle tone, hyperthermia, diarrhea, yawning, nasal stuffiness, and sneezing (Kraft et al. 2016). The presentation of withdrawal symptoms in the newborn varies from mild to severe, thus the diagnosis of NAS is based on the cardinal signs of withdrawal (Kraft et al. 2016). Assessments of observed behaviors are completed every 3–4 h, most commonly using the 31 item-scale Finnegan scoring instrument, and the total score is determined to assess the presence and severity of symptoms and the need for pharmacologic intervention, dosing, and weaning (Kraft et al. 2016). The drugs or medicines that can lead to NAS include: opioids, methadone, alcohol, selective serotonin reuptake inhibitors, benzodiazepines, and nicotine (American College of Obstetricians and Gynecologists [ACOG] 2017). Cannabis use during pregnancy is also increasing in the U.S., especially during the first-trimester when the fetus is most susceptible (Alshaarawy and Anthony 2019). Smoking cannabis during pregnancy has been linked to lower birth weight newborns, but there is limited evidence that cannabis-exposed newborns have complications that require a Neonatal Intensive Care Unit (NICU) admission (National Academies of Sciences, Engineering, and Medicine 2017). Further effects of cannabis-exposed newborns are not clear due to lack of research.

Newborn complications from maternal substance use often cause a prolonged LOS in a higher cost hospital bed (Lee et al. 2015). NAS has increased from 1.5 to 6.0 per 1000 births from 1999 to 2013, and has been related to a significant increase of hospital charges annually (ACOG 2017; McQueen et al. 2015). Ultimately, the financial responsibility to care for these patients is then placed on the hospital and can lead to a financial burden. The impact of substance use by pregnant women using medicinal or recreational cannabis, prescription drugs, and/or illicit drugs needs further study.

## Methods

The purpose of this study was to determine the impact of substance use by pregnant women (from admission dates of January 1, 2013 to December 31, 2017) on a Colorado hospital in a community where recreational cannabis has been legalized. The Institutional Review Board (IRB) granted approval for the study. Data were gathered retrospectively through the existing electronic health record (EHR) at an acute-care, inpatient facility, and did not involve interaction with the patients. A sequence was written for the EHR system, and the outcome measures listed below were extracted through the program. An additional source of statewide data to include the characteristics of a normal healthy newborn and a neonate with health concerns groups were used for comparison. There is no standard for NAS hospital coding (NIDA 2020); therefore, the NAS comparison was to a neonate with health concerns. The de-identified datasets described mothers and newborns who were located on the Maternity Unit who tested positive for drugs: opioids, cocaine, cannabis, barbiturates, benzodiazepines, amphetamines, and methadone. Determination of which mothers and newborns were screened was at the discretion of the healthcare providers, and was determined by self-report of drug use, or presence of NAS withdrawal symptoms in the newborn. Mothers and newborns who tested negative for drugs, or who were not drug screened, were excluded from the consideration.

The datasets were analyzed separately using the following outcome measures: positive substance use result from a urinalysis or blood analysis for the mother at the time of delivery, and positive substance result in the urine, meconium, or cord blood for newborn population. The data were analyzed according to health insurance; newborn length of stay (LOS); newborn disposition upon discharge; cost of newborn bed per day according to bed placement at time of discharge; and total number of positive tests for newborn and maternal populations. Descriptive data were analyzed and for several variables separated by year to include age, race, ethnicity (Table 1); and type of admission and insurance (Table 2) for mothers; and birthweight, gestational age, and length of stay for newborns (Table 3).

**Table 1:** Demographic characteristics of mothers included in study

Demographic	Cohort	Count (Percentage)
Age	14–19	48 (4.7%)
	20–24	215 (21.0%)
	25–29	204 (19.9%)
	30–34	98 (9.6%)
	35–39	35 (3.4%)
	40–49	7 (0.7%)
Race	American Indian / Alaska Native	18 (3.0%)
	Asian	1 (0.2%)
	Black or African American	16 (2.6%)
	Hawaiian	8 (1.3%)
	Hispanic	44 (7.3%)
	White	422 (69.5%)
	Refused to answer	26 (4.3%)
	Unknown	72 (11.9%)
Ethnicity	Hispanic	216 (35.6%)
	Not Hispanic	296 (48.8%)
	Refused to answer	78 (12.9%)
	Unknown	17 (2.8%)

**Table 2:** Mothers’ admission type and insurance type by year of admission

Year	2013	2014	2015	2016	2017
Number of participants	78	91	114	130	194
Type of admission					
Clinical	39.7%	29.7%	36.0%	32.3%	33.0%
Inpatient	59.0%	62.6%	61.4%	65.4%	64.4%^b^
Observation	1.3%	7.7%	2.6%	2.3%	2.6%
Type of insurance					
Public^a^	90.5%^c^	91.2%	92.1%	93.1%	90.7%
Private	3.2%	5.5%	7.0%	7.0%	7.7%
Self-pay	6.3%	3.3%	1.0%	0%	1.5%

Clinical and Outpatient status – not formally admitted to the hospital, anticipated stay less than 48 h at provider’s discretion

Inpatient status – formally admitted to the hospital, anticipated stay greater than 2 midnights at provider’s discretion (CMS 2018)

^a^ No statistically significant change in the use of public health insurance occurred as determined by the Cochran-Armitage chi-square test (*χ*^2^ = 0.002, df = 1, *p* = 0.96)

^b^ A single patient whose type of admission was listed as Surgical Day Care has been added to the Inpatient group

^c^ The type of insurance was not reported for 15 participants in 2013 and the percentages for this year were found only for the other 63 participants

**Table 3:** Characteristics of newborns by the drug category

Type of drug	Birthweight, mean ± sd^a^, grams	Gestational age, mean ± sd, days	Length of stay, mean ± sd, days^b^
Opioids	2792 ± 15 (62)	265.5 ± 14.6 (53)	17.4 ± 25.5 (73)
Cocaine	2745 ± 18 (4)	265.0 ± 17.8 (3)	4.8 ± 6.4 (5)
Cannabis	2872 ± 12 (189)	269.1 ± 12.2 (182)	4.6 ± 9.5 (199)
Barbiturates	3255 ± 12 (2)	264.5 ± 12.0 (2)	1.5 ± 0.7 (2)
Benzodiazepines	2883 ± 10 (3)	257.0 ± 9.9 (2)	3.0 ± 1.0 (3)
Amphetamines	2909 ± 20 (18)	262.0 ± 20.0 (15)	5.6 ± 6.2 (21)
Methadone	3011 ± 10 (18)	273.1 ± 9.8 (21)	16.5 ± 17.3 (23)
Multiple drugs	2710 ± 17 (82)	264.3 ± 17.1 (71)	52.6 ± 62.8 (93)

The number of newborns for whom the data was obtained is shown in parentheses

^a^ *sd* Standard deviation

^b^ The average length of stay among the newborns exposed to cannabis was significantly shorter than that in the opioid (*t* = 8.22, df = 222, *p* < 0.001), amphetamine (*t* = 6.57, df = 95, *p* < 0.001), and methadone (*t* = 5.87, df = 47, *p* < 0.001) groups as shown by two-sample *t*-tests of log-transformed LOS values. No other comparisons of length of stay were significant

Cochran-Armitage chi-square testing was completed to test for trends in categorical data proportions and evaluate the proportions of screened patients who tested positive for drugs, including medical or recreational cannabis, since the data appeared to indicate an increasing trend in these proportions over the years (from 2013 to 2017). The Cochran-Armitage test was also used to determine the trends in the use of public health insurance and disposition of newborns to another acute care hospital or home. Chi-square tests were implemented to compare the proportions of newborns who tested positive to drugs and evaluate the associations between the drugs and newborn hospital accommodations.

In the analysis of the LOS, the significant differences among the groups were identified with the use of two-sample *t*-tests. The comparisons were carried out by the type of drug. Prior to the implementation of the tests, the data were log-transformed due to considerable skewness in the distribution of LOS. The Bonferroni adjustment of the significance level was taken into consideration to achieve the 5% familywise error rate among all 21 comparisons of individual drugs. For the comparison of the LOS, all newborns were separated into two groups: those who only tested positive for cannabis (cannabis-exposed) and those who tested positive for a single drug different from cannabis (other-drug exposed). Ninety-three newborns who tested positive for two or more drugs out of the total number of 419 were excluded from the comparisons of specific drugs. The dynamic of the LOS over time was analyzed using the linear regression methodology. Once again, LOS values were log-transformed, while the time was represented by years, 2013–2017. A first-order linear regression model was fit to evaluate the significance of the linear trend. Regression-based confidence intervals for the estimated average LOS were obtained for 2013 and 2017 to evaluate the significance of the increase in LOS.

Total costs for the healthcare system were calculated based on the cost for a normal newborn bed (NSY) of $2570.00 per day and $3560.00 per day for a SCNSY (special care nursery) bed at the Colorado community hospital, observed LOS among the cannabis-exposed and other-drug exposed newborns, and the number of newborns in each category. Cost information was not available for the pediatric (PEDS) bed placement, a moderate acuity/ intervention type of bed.

## Results

The de-identified dataset analyzed included 607 mothers and 419 newborns (total *N* = 1026) who tested positive for drugs. Demographic data were only available for those who tested positive for drugs (see Table 1). Out of the total mothers and newborns (1026) who tested positive for drugs, 224 (21.8%) tested positive for more than one substance on the drug screen. Out of the 224 who tested positive for multi-substance use, 112 of these (50%) were cannabis plus another drug (see Table 4).

**Table 4:** Results of drug testing by year of admission

Year	2013	2014	2015	2016	2017
Total number of patient visits on Maternity Unit	4952	5035	4986	5112	4930
Total number of toxicology screens performed	308	334	411	517	640
Total participants who were screened and tested positive to drugs	103	149	208	245	321
Percentage of screened patients who tested positive to drugs^a^	33.4%	44.6%	50.6%	47.4%	50.2%
Participants tested positive to multi-substances	17	39	38	58	72
Percentage of those testing positive to drugs who tested positive to multi-substances	16.5%	26.2%	18.3%	23.7%	22.4%
Participants tested positive to multi-substances, including cannabis	7	24	18	34	29
Percentage of multi-substance which included cannabis	41.0%	61.5%	47.0%	58.6%	40.3%

The results include the drug testing for both mothers and newborns

^a^ The increase in the percentage of patients who tested positive over the years is statistically significant (*χ*^2^ = 17.93, df = 1, *p* < 0.001) using the Cochran-Armitage test for a trend in proportions

### Trends in drug use proportions over the years

The proportion of patients testing positive for drugs (Table 4) increased significantly between 2013 (33.4%) and 2017 (50.2%) (*p* < 0.001), while the proportion of mothers using public insurance did not change significantly over this period (Table 2). Between 2013 and 2017, the proportion of newborns testing positive for cannabis increased significantly (*p* < 0.001), the proportion testing positive for benzodiazepines decreased significantly (*p* < 0.001), and the proportions testing positive for opioids (*p* = 0.17), cocaine (*p* = 0.26), amphetamines (*p* = 0.13), or methadone (*p* = 0.95) did not change significantly (Table 5). The number of barbiturate-positive tests was too small to allow evaluation. The proportions of mothers using certain drugs decreased significantly for cocaine (*p* = 0.04) and benzodiazepines (*p* < 0.001), increased significantly for methadone (*p* < 0.01), and the proportions of those testing positive for opioids (*p* = 0.30), cannabis (*p* = 0.12), or amphetamines (*p* = 0.67) did not change significantly (Table 6). The number of barbiturate-positive tests was too small to allow evaluation.

**Table 5:** Newborn exposure to drugs by years

Year	2013	2014	2015	2016	2017
Total^a^, including	25	58	94	115	127
Opioids	12	22	32	31	43
Cocaine	2	4	2	5	4
Cannabis	5	18	58	84	80
Barbiturates	0	1	0	0	2
Benzodiazepines	3	4	2	2	0
Amphetamines	6	14	13	23	17
Methadone	0	12	7	6	16

The trends in proportions were determined according to the Cochran-Armitage chi-square test

The proportion of newborns testing positive for cannabis increased significantly (*χ*^2^ = 26.76, df = 1, *p* < 0.001), the proportion testing positive for benzodiazepines decreased significantly (*χ*^2^ = 13.95, df = 1, *p* < 0.001), and the proportions testing positive for opioids (*χ*^2^ = 1.91, df = 1, *p* = 0.17), cocaine (*χ*^2^ = 1.30, df = 1, *p* = 0.26), amphetamines (*χ*^2^ = 2.35, df = 1, *p* = 0.13), or methadone (*χ*^2^ = 0.004, df = 1, *p* = 0.95) did not change significantly. The number of barbiturate-positive tests was too low to allow evaluation

^a^ The totals are not equal to the sums of separate drug categories, since more than one drug could be found at the same time

**Table 6:** Mothers’ exposure to drugs by years

Year	2013	2014	2015	2016	2017
Total^a^, including	78	91	114	130	194
Opioids	26	34	39	45	57
Cocaine	6	3	2	3	4
Cannabis	38	53	74	80	119
Barbiturates	0	1	0	2	1
Benzodiazepines	10	5	2	1	5
Amphetamines	14	17	12	22	30
Methadone	1	12	4	9	27

The trends in proportions were determined according to the Cochran-Armitage chi-square test

The proportions of mothers using particular drugs decreased significantly for cocaine (*χ*^2^ = 4.24, df = 1, *p* = 0.04) and benzodiazepines (*χ*^2^ = 13.60, df = 1, *p* < 0.001), increased significantly for methadone (*χ*^2^ = 6.78, df = 1, *p* = 0.009), and the proportions of those testing positive for opioids (*χ*^2^ = 1.06, df = 1, *p* = 0.30), cannabis (*χ*^2^ = 2.46, df = 1, *p* = 0.12), or amphetamines (*χ*^2^ = 2.35, df = 1, *p* = 0.67) did not change significantly. The number of barbiturate-positive tests was too small to allow evaluation

^a^ The totals are not equal to the sums of separate drug categories, since more than one drug could be found at the same time

### Determining impact to healthcare at a Colorado community hospital

The average LOS of newborns showed a significant linear trend (*p* < 0.001) over the years 2013 to 2017 (Table 7). Determined with 95% confidence, the estimated average LOS was between 2.48 and 4.58 days in 2013 and between 6.29 and 9.30 days in 2017 indicating a significant increase in LOS. The average LOS among the cannabis-exposed newborns was significantly shorter than that in the opioid, amphetamine, and methadone groups (all *p*-values below 0.001) (Table 3). No other LOS comparisons among individual drug groups were significant. There were 199 newborns who tested positive for cannabis (cannabis-exposed) and 127 who tested positive for a single other drug (other-drug exposed). The average LOS (± sd) for the cannabis-exposed group was 4.6 ± 9.5 days and for other-drug exposed 14.2 ± 21.4 days, a highly significant difference (*p* < 0.001). The average LOS (± sd) for the cannabis-exposed group was 4.6 ± 9.5 days and for other-drug exposed 14.2 ± 21.4 days, a highly significant difference (*p* < 0.001).

**Table 7:** Newborn length of stay by years

Year	2013	2014	2015	2016	2017
Number of newborns	25	58	94	115	127
Length of stay, days (mean ± sd)	4.3 ± 5.2	10.9 ± 23.3	15.2 ± 23.9	19.2 ± 38.2	25.5 ± 50.4

The average length of stay of drug-exposed newborns increased significantly over years as shown by the correlation analysis of log-transformed LOS values versus the time (*p* < 0.001)

The proportions of newborns in SCNSY or PEDS beds were higher than the respective proportions in NSY beds among the newborns testing positive to opioids (*p* < 0.001), benzodiazepines (*p* < 0.01), amphetamines (*p* < 0.001), and methadone (*p* < 0.001). Conversely, the proportion of SCNSY/PEDS cannabis-exposed newborns was lower (*p* < 0.001) than the corresponding proportion in the NSY group (see Table 8). The two groups did not differ significantly on the proportions with respect to cocaine or barbiturates.

**Table 8:** Bed placement of newborns by drug type

	SCNSY or PEDS^a^	NSY^b^
Opioids**	107 (54)	33 (19)
Cocaine	7 (1)	10 (4)
Cannabis**	74 (46)	171 (153)
Barbiturates	0 (0)	3 (2)
Benzodiazepines*	10 (2)	1 (1)
Amphetamines*	48 (11)	25 (10)
Methadone**	34 (19)	7 (4)

According to chi-square tests for equality of proportions, the proportion of newborns in SCNSY/PEDS placement was higher than that in the NSY placement among the opioid-exposed newborns (*χ*^2^ = 68.86, df = 1, *p* < 0.001), benzodiazepine-exposed newborns (*χ*^2^ = 6.84, df = 1, *p* < 0.01), amphetamine-exposed newborns (*χ*^2^ = 10.95, df = 1, *p* < 0.001), methadone-exposed newborns (*χ*^2^ = 21.33, df = 1, *p* < 0.001), lower among the cannabis-exposed newborns in (*χ*^2^ = 69.07, df = 1, *p* < 0.001), and did not differ significantly among the cocaine-exposed newborns (*χ*^2^ = 0.08, df = 1, *p* = 0.78). The number of barbiturate-positive tests was too small to allow evaluation

*Abbreviations*: *SCNSY* Special care nursery – high level acuity and intervention, *PEDS* Pediatric – moderate acuity and intervention, *NSY* Normal newborn nursery – low level acuity and intervention. The number of those exposed to a single particular drug is shown in parentheses

* and ** indicate statistically significant differences between the proportions of SCNSY/PEDS and NSY newborns exposed to specific drugs with *p* < 0.01 (*) and *p* < 0.001 (**), respectively, as described above

^a^ 66 newborns placed in SCNSY or PEDS were exposed to multiple drugs

^b^ 27 newborns placed in NSY were exposed to multiple drugs

The proportion of newborns discharged to other acute care hospitals decreased over time (*p* < 0.001), the proportion discharged to home w/home care did not change significantly (*p* = 0.98), and the proportion discharged home increased over time (*p* < 0.001) (see Table 9).

**Table 9:** Newborn disposition by year

Year	2013	2014	2015	2016	2017
Home	8	23	56	94	112
Acute Care Hospital	15	30	14	0	0
Home w/Home Health	0	4	22	20	9
Other	2	1	2	1	6
Total	25	58	94	115	127

The trends in proportions were determined according to the Cochran-Armitage chi-square test

The proportion of newborns discharged home increased significantly (*χ*^2^ = 71.58, df = 1, *p* < 0.001), the proportion discharged to another acute care hospital decreased significantly (*χ*^2^ = 125.11, df = 1, *p* < 0.001), and the proportions discharged to home w/home health did not change significantly (*χ*^2^ = 0.001, df = 1, *p* = 0.98)

Taking into account the number of cannabis-exposed and other-drug exposed newborns (Table 8), the costs of NSY and SCNSY beds at $2570.00 per day and $3560.00 per day, respectively, as well as the LOS of newborns in each category (Table 10), the total costs to the Colorado community hospital were calculated at $2,885,139 for the 199 cannabis-exposed and $23,495,221 for the 220 other-drug exposed newborns. The comparable costs of caring for the same numbers of healthy newborns would be $1,029,029 and $1,137,620, respectively. The cannabis-exposed newborns are still more costly to the community hospital when compared to a normal healthy newborn. These additional costs equated to $1,856,110 (cannabis-exposed, *p*-value < 0.001) and $22,357,601 (other-drug exposed, *p*-value < 0.001).

**Table 10:** Newborn average length of stay and total hospital charge by type of bed placement

Type of Charge	Average Length of Stay for Newborns ± sd^a^	Total Cost of Hospital Stay for a Newborn
Normal Newborn	2.6 ± 1.2 days (only cannabis)	$6618.17
SCNSY	11.4 ± 18.3 days (only cannabis)	$40,584.00
Normal Newborn	5.2 ± 6.2 days (other drugs)	$13,364.00
SCNSY	41.5 ± 53.5 days (other drugs)	$147,751.64

*Abbreviation*: *SCNSY* Special care nursery

^a^ The average length of stay of cannabis-exposed newborns was significantly shorter than that of other-drug exposed newborns according to a two-sample *t*-test of log-transformed values (*t* = − 5.79, df = 185, *p* < 0.001)

## Discussion

The proportion of patients who tested positive for drugs increased significantly from 2013 to 2017, correlating with the increasing trend of substance use that has become a “2016-2020 Maternal and Child Health” priority in the state of Colorado (Colorado Department of Public Health and Environment [CDPHE] 2019). The reported cases of NAS in Colorado in 2018 were 5.4 per 1000 hospital births (National Institute on Drug Abuse [NIDA] 2020). The average LOS in Colorado for a neonate with health concerns appeared to be slightly decreasing where the average LOS was 2.4 days in 2013 and 2.3 days in 2017 (Colorado Hospital Association [CHA] undated). When comparing LOS to trends in CO, the results of this study differ where the other-drug exposed newborns LOS was trending upward. These newborns were staying longer in the hospital, creating higher costs, which then impacts the Colorado community hospital. The increasing LOS dynamic for drug-exposed newborns held true even after the logarithmic data transformations that minimized the influence of high outliers largely responsible for the five-fold increase in average LOS reported in Table 7.

This study showed that a significant number of those testing positive for more than one substance included cannabis as one of the drugs. From 2002 to 2017, cannabis use during pregnancy in the U.S. was increasing, and approximately 19% of pregnant woman use of cannabis was related to cannabis dependence (Alshaarawy and Anthony 2019). In Colorado, 14.6% of mothers used cannabis during pregnancy from 2015 to 2017 (CDPHE 2019). Other research reveals that cannabis use during pregnancy, especially when combined with other substances, may adversely affect neonatal behavior (Ryan et al. 2018). The resulting LOS in this study among the cannabis-exposed newborns is significantly shorter than that in the opioid, amphetamine, and methadone groups. Even though the LOS for cannabis-exposed newborns was found to be shorter, it is still significantly longer than the average 1.9-day LOS in Colorado for a normal, healthy newborn and the 2.3 days for a neonate with health concerns (CHA undated). A possible explanation for the increased LOS could be the limited research available to health care providers on the effects of cannabis-exposure (National Academies of Sciences, Engineering, and Medicine 2017; Warshak et al. 2015). The newborns may have stayed longer in the hospital to allow for additional observation due to a positive drug screen.

The type of newborn bed placement affects the Colorado community hospital due to an increased cost for a SCNSY bed. Cannabis-exposed newborns had a statistically significant higher rate of normal NSY placement, whereas the other drug-exposed newborns had a significantly higher rate of placement in a SCNSY or PEDS bed (see Table 8). This suggests that cannabis-exposed newborns are of lower acuity, and therefore less costly to the healthcare system, as compared to the other drug-exposed newborns. Cannabis-exposed newborns placed in a normal newborn bed cost approximately $6618.17 per person when calculated with their average LOS of 2.6 days, however, this was still greater than the average cost of $5171 for a normal newborn hospital stay in the state of Colorado (CHA undated). This study revealed results similar to previous studies where a significant financial impact is occurring for NAS newborns. The costs grow substantially when newborns are assigned to SCNSY and the LOS is longer. NAS has increased where newborn LOS is longer and led to $1.5 billion of hospital charges annually which is taxing on “an already overburdened health care system” (McQueen et al. 2015, p. 1763).

Mothers testing positive for drugs were more likely to have public health insurance, including Medicaid, than other types of insurance (Table 2). The higher rate of public insurance coverage among drug-exposed newborns might contribute to lower reimbursement to the Colorado community hospital, thus increasing the cost burden. Reimbursement rates to the hospitals, or Medicaid payments, in many states do not meet the average healthcare costs for these patients (Harrison 2016). This correlates with other research findings where the “mean hospital charge for infants diagnosed with NAS was $53,400.00, and 77.6% of charges for NAS were attributed to state Medicaid programs” (Lee et al. 2015, p. 396).

It has been shown that prematurity or low birthweight could potentially cause an increased LOS for newborns (Eneriz-Wiemer et al. 2018). However, in different drug groups analyzed in this study, the average birthweight of newborns ranged from 2710 to 3255 g and the average gestational age ranged between 257 and 273 days (see Table 3). Taking into account the standard errors, these figures do not constitute a significant difference from those considered normal for newborn birthweight (between 2500 and 4000 g at gestational term) and gestational age (between 259 and 280 days) (ACOG 2020; KidsHealth 2020). Thus, we do not attribute the extended LOS for drug-exposed newborns to either prematurity or low birthweight.

The disposition of the newborn can impact healthcare systems if newborns continue to need healthcare after being discharged from the Colorado community hospital. The results show that the proportion of newborns discharged directly to home increased over time, while the proportion of newborns discharged to another acute care hospital decreased (Table 9). The test also did not reveal any trend in the proportions of newborns discharged to home with home care. The results showed that the increased financial responsibilities remained within the Colorado community hospital. This can cause an impact to other healthcare facilities since they are now having a decreased number of these newborns being transferred to their facilities for healthcare needs.

### Limitations

This study has several limitations. Having data of mothers and newborns living only in a community in Colorado decreases the ability to generalize the results of this study to a larger population. The de-identified data for the mothers included three admission types: clinical, observation and inpatient status. Mothers who are giving birth are admitted with an inpatient status, but those with clinical or observation status are sent home at the provider’s discretion. Therefore, some of the mothers may have been admitted more than once during the study period. This could explain the larger number of mothers testing positive for drugs as compared to the newborns. The retrospective design eliminated the researchers’ control of variables, such as determining whether a drug screen would be collected, which was decided by the healthcare providers’ judgement during the hospital stay.

Mothers who did not admit to drug use and newborns who did not display withdrawal symptoms during the hospital stay were not tested. This may have resulted in an underestimation of drug use, which would decrease the study sample size. The contributors from the hospital revealed that since the legalization of recreational cannabis in 2014, some healthcare providers did not perform a drug screen on patients who admitted to either recreational or medicinal cannabis use. These patients were not included in the study datasets since they did not have a positive drug-screen result. This exclusion could affect the trend in data, increasing the respective number of positive drug screens in the first year of the study data (2013) compared to the last 4 years of the study data (2014–2017).

Newborns exposed to only cannabis may not display the commonly found signs of withdrawal found in NAS, so may not have been tested. This would result in a decreased detection of cannabis only use during pregnancy, thereby decreasing the study sample size and creating a bias toward those utilizing other substances. Newborns exposed to opiates in utero may not display typical signs of NAS until after they are discharged from the hospital, which would decrease the numbers of opiate-exposed newborns tested and included in the study. The data available on bed placement was the location of the newborn at discharge; therefore, the cost calculation was an estimation based off of LOS. The PEDS bed placement was not included in the cost calculation due to the unavailability of that data to the researchers. There was no documentation on the frequency of drug use and whether drugs were used throughout all three trimesters of pregnancy, which could affect whether the newborn exhibited signs of withdrawal. The newborn LOS could have been affected by other medical or social factors, or by method of delivery, which were not included in this study. The data included in the study described mothers and newborns who met the inclusion criteria for this study, testing positive on a drug screen. Although the number of those who had a drug screen with a negative result is known (see Table 4), the variables of interest on these patients were not available and the characteristics of the specific lab tests were also not obtained.

## Conclusion

This study found that drug-exposed newborns had a substantial financial impact on a Colorado community hospital, largely from more expensive bed placement and longer LOS than for healthy newborns. This effect was less pronounced for cannabis-exposed newborns than for those exposed to other-abused drugs. All of these factors could cause a significant impact to healthcare systems due to lower reimbursement rates when these newborns qualify for public health insurance.

A recommendation for healthcare providers would be to identify substance use during the prenatal period by routinely screening for substance use by utilizing validated questionnaires, having candid conversations with the patients, or objective measures such as urine drug screens (Ryan et al. 2018). Once substance use is identified during the pregnancy, counseling abstinence and referral for treatment should be implemented (Ryan et al. 2018). Identification of maternal substance use will better identify which neonates may have health complications requiring an increased LOS at birth. A recommendation to address drug-exposed newborn LOS would be inpatient/outpatient newborn treatment to assist in reducing costs to hospitals.

Due to limited research, intrauterine substance exposure and the long-term impact to the exposed child should be further researched. Future studies should include multiple settings and locations across the U.S. to incorporate a larger sample size and increase generalizability, type of substance use, frequency of use, route of administration, which trimester of pregnancy the drug use occurred in, and additional medical or social factors which could contribute to an increased newborn LOS and healthcare costs. As social and political changes are made regarding cannabis legalization in the U.S., more research is also needed on newborn exposure during pregnancy to allow for healthcare providers to deliver safe, quality care to these patients.

## Data Availability

Even though the researchers only had access to anonymized data, the IRB denied us the right to share the data. It is unknown why the IRB denied this request.

## Appendix

### Variables extracted from the EHR

- Dates of service
- Admission date
- Initial location
- Reason for visit
- Zip code
- Insurance type
- Gender
- Age
- Ethnicity
- Race
- Admission type
- Newborn birthweight
- Newborn gestation age
- Discharge disposition
- Discharge date
- Discharge location
- Total LOS
- Newborn diagnosed with NAS
- Drug test code
- Drug positive for

An additional dataset included:

- Summary of the total number of patient visits on this unit per year
- Total number of toxicity screens that were performed
- Percentage of positive toxicity screens
- Number of positive toxicity screen
- Discharge disposition of percentage d/c home
- Total of NAS diagnoses

## Abbreviations

ACOG: American College of Obstetricians and Gynecologists
CDPHE: Colorado Department of Public Health and Environment
CHA: Colorado Hospital Association
EHR: Electronic health record
IRB: Institutional Review Board
LOS: Length of stay
NIDA: National Institute on Drug Abuse
NAS: Neonatal Abstinence Syndrome
NICU: Neonatal Intensive Care Unit
NSY: Normal newborn nursery
PEDS: Pediatric
SCNSY: Special care nursery
U.S.: United States
